# Joint external evaluation of the international health regulations (2005) capacity in South Sudan: assessing the country´s capacity for health security

**DOI:** 10.11604/pamj.supp.2022.42.1.33842

**Published:** 2022-06-09

**Authors:** Argata Guracha Guyo, Kibebu Kinfu Berta, Otim Patrick Ramadan, Malick Gai, Alice Igale Lado, Gabriel Thuou Loi, Mathew Tut Kol, Mary Denis Obat, Sylvester Maleghemi, Fabian Ndenzako, Olushayo Oluseun Olu

**Affiliations:** 1 World Health Organization, WHO Country Office, Juba, South Sudan,; 2 World Health Organization, East and South Africa, Sub-Regional Office, Nairobi, Kenya,; 3 Ministry of Health, Juba, South Sudan

**Keywords:** International health regulation, joint external evaluation, health security, South Sudan

## Abstract

**Introduction:**

joint external evaluation is a voluntary and collaborative process to assess a country´s capacity under International Health Regulations (2005) to prevent, detect, and respond to public health threats. The main objective is to measure a country´s status in building the necessary capacities to prevent, detect, and respond to infectious disease threats and establish a baseline measurement of capacities and capabilities. The Republic of South Sudan conducted the Joint External Evaluation from 16-20 October 2017, where its capacities were assessed to public health threats per the International Health Regulation (2005).

**Methods:**

cross-sectional descriptive study of the Joint External Evaluation process and the findings are described along with major findings and recommendations for the country.

**Results:**

South Sudan’s overall mean score across 48 indicators was 1.5 (min= 1, max= 4) and 42/48 indicators (87.5%) scored < 2 on a 1 to 5 scale. Technical areas in the prevent category with the lowest score were antimicrobial resistance, biosafety and biosecurity, and National legislation, policy, and financing. In the detect category, the mean score was 2. Technical areas with the lowest mean scores were workforce development and the National Laboratory System. Preparedness, medical countermeasures, personnel deployment, linking public health, and security authorities had the lowest scores in the respond category. Chemical events, radiation emergencies, and points of entry had a score of 1 in the other IHR-related hazards and points of entry category.

**Conclusion:**

South Sudan’s mean score of 1.5 can be attributed to several civil conflicts experienced, which have impacted negatively on the health system. Recommendations from the Joint External Evaluation need to be implemented and these must be aligned with the costed National Action Plan for Health Security.

## Introduction

Between 2001 and 2021, several epidemics and pandemics have posed a severe threat to global health security. These epidemics and pandemics include Severe Acute Respiratory Syndrome (SARS), avian influenza subtypes (H5N1 and H7N9) in Southeast Asia and China, Middle East Respiratory Syndrome (MERS) in the Middle East and South Korea, Ebola virus disease (EVD) in West Africa. In addition, most recently, coronavirus disease 2019 (COVID-19) was first detected in China and has spread to every country in the world. These infectious diseases are mainly driven by the emergence and spread of new pathogens, globalization of travel, food, medicines, antimicrobial resistance rise, and accidental spillover of biohazard agents [1]. The morbidity, mortality, and economic impact of such public health threats can be enormous [2,3]. For example, COVID-19 has resulted in 260 million confirmed cases, and over 5 million deaths as of November 30th, 2021, with a contraction of the global economy, experienced during the recession 80 years ago [4-6].

In the World Health Organization (WHO) Africa Region (AFRO), an acute public health event (PHE) occurs every 3-4 days totalling more than 150 PHEs every year, putting the entire region at high risk of health security threats [7,8]. Some countries like South Sudan face a double jeopardy of PHEs and prolonged armed conflicts. South Sudan has witnessed a protracted humanitarian crisis triggered by an armed conflict that erupted in 2013, disrupted the country’s health system, livelihood, and economy [8]. This has resulted in a weak health system with low immunization coverage (i.e. pentavalent-3 coverage was 45% in 2019), leading to an outbreak of vaccine-preventable diseases such as measles in 2018 [9].

WHO member states drafted the international health regulation (IHR 2005) which was adopted by the World Health Assembly (WHA) in 2005 based on the lessons learnt from the first pandemic of the 21st century caused by SARS [10]. The IHR (2005) mandates all countries to develop and strengthen their core capacities to prevent, detect, assess, report, and respond to public events and other hazards [11]. The IHR (2005) came into force in 2007, with all signatory countries given five years to develop the core capacities of IHR (2005). As of 2014, only 30% of states parties had met the required capabilities. In 2015, WHO recommended that “countries move from exclusive self-assessment to approaches that combine self-evaluation, peer review, and voluntary external evaluations involving a combination of domestic and independent experts” [12,13]. In that perspective, WHO developed the joint external evaluation (JEE) process and the JEE tool in February 2016 as part of the IHR (2005) monitoring and evaluation framework to determine countries´ capacity to prevent, detect, and respond to public health threats [14,15]. The JEE was conducted in South Sudan from 16-20 October 2017 [16]. The objective of this study is to document the country´s capacities to prevent, detect and respond to public health threats per the IHR (2005) core capacities. The findings guided the development of the post-JEE costed national action plan for health scurity (NAPHS) [17].

## Methods

**Study design and area:** we conducted a cross-sectional descriptive study of the JEE processes conducted for South Sudan in Juba. The JEE was performed using the WHO guidelines and JEE tool. It consists of 19 technical areas structured into four main categories and 48 questions/indicators (prevent, detect, respond, and other IHR-related hazards and points of entry (PoE). The 19 technical areas comprise 48 indicators that are measured by scale criteria ranging from 1 to 5 (1 = no capacity, 2 = limited capacity, 3 = developed capacity, 4 = demonstrated capacity, and 5 = sustainable capacity) [5] (Table 1). The scores are represented by different colors 1= red, 2 and 3= yellow, and 4 and 5= green [13] (Table 1). Furthermore, the 19 technical areas were categorized in four areas: prevent, detect, respond, and IHR related hazards and points of entries. The first stage of the evaluation is an internal self-assessment completed by the country using self-reported data for the various indicators on the JEE. In South Sudan, this was done from 16 to 20 October 2017. Before the implementation of the JEE, a 1-week consultative meeting was conducted. The phase one process began with the setup of a multi-disciplinary and multi-sectoral team of 25 persons comprising technical departments among key ministries (e.g. health, humanitarian affairs, justice, animal resource and fisheries, environment and forestry, agriculture, wildlife and tourism, petroleum, immigration, civil aviation authority, food and drugs authority. The team comprised nine experts from different institutions such as WHO, the Food and Agriculture Organization (FAO), and different countries, including Nigeria and the United Republic of Tanzania. External subject-matter experts were identified with support from WHO AFRO, and the country´s internal evaluation report was shared with them. Before coming into the country, the team reviewed this self-assessment data, which provided a baseline understanding of the country´s health security capabilities.

**Table 1:** joint external evaluation core capacity technical areas and scores

Thematic area	Technical areas	Number of indicators
Prevent	National legislation, policy and financing	2
	IHR coordination, communication and advocacy	1
	Antimicrobial resistance	4
	Zoonotic diseases	3
	Food Safety	1
	Biosafety and biosecurity	2
	Immunization	2
Detect	Workforce development	3
	National laboratory system	4
	Real-time surveillance	4
	Reporting	2
Respond	Preparedness	2
	Emergency response operations	4
	Linking public health and security authorities	1
	Medical countermeasures and personnel deployment	2
	Risk communication	5
Other IHR related hazards and PoEs	Points of entries	2
	Radiation emergencies	2
	Chemical events	2

Source: joint external evaluation tool: international health regulations - SCORE for health data

There was a one-week workshop from 16-20 October 2017 that facilitated in-depth discussion of the self-reported data and structured site visits to Juba international airport and Nimule land crossing border points. They have joined hands with an 25 national teams of experts, including other identified government agencies, none-governmental organizations (NGOs), and united nations (UN) agencies. The external assessment team reviewed the self-assessment report and associated reference documents; discussed their observations and questions with the national experts; conducted site visits at Nimule land crossing in Eastern Equatoria state and Juba International Airport (JIA), and assigned scores to each of the 48 indicators following consensus with the team of national experts. After conducting the evaluation visit, the evaluation team drafted a report that identified status levels for each indicator and analyzed the country´s capabilities, gaps, opportunities, and challenges. The report was shared with the ministry of health (MoH). In addition, with permission from the MoH, the report was shared among various stakeholders. The objective of sharing the report was to facilitate support to implement identified best practices, address challenges, develop monitoring, accountability and evaluation tools.

**Data analysis and presentation:** the South Sudan JEE scores for the 19 technical areas were analyzed using microsoft excel for descriptive statistics. The overall mean score of the South Sudan score was then calculated.

**Ethical clearance and approval:** administrative clearance for this study was provided by the ministry of health of South Sudan. Moreover, the Research Ethics Review Board of Ministry of Health provided clearance for the publication of manuscript under (MoH/RERB/D.03/2022) clearance number. Besides, WHO provided executive clearance for the publication of the manuscripts (WHO ePub-IP-00331327-EC).

## Results

**Figure 1:**
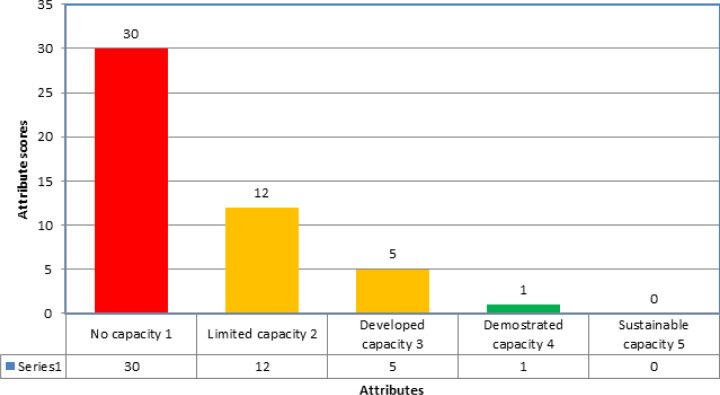
number of indicators per score during the JEE, October 17-20 the Republic of South Sudan

On a scale of 1-5 overall mean score for the 48 indicators in the 19 technical areas was 1.5 (no to limited capacity). Out of 48, 30 (63%) showed the country has no capacity under the IHR (2005) requirement for health security. A total of 42 out of 48 indicators (88%) scored under the no capacity and limited capacity category. Figure 1 illustrates the combined indicators score in the country. In the prevent category, seven technical areas with 15 indicators were included during the JEE. The mean score was again 1.5 (no to limited capacity). Nine out of 15 (60%) indicators had a 1 (no capacity) score. Technical areas with the lowest mean scores were antimicrobial resistance (AMR) and biosafety and biosecurity with a score of 1 each (no capacity) (Table 2). The indicator scores ranged from 1.3 to 3 in the Detect category, with 11 of the 13 indicators (86%) in 4 technical areas having a score ≤ 3. The mean score was 2 (limited capacity), and technical areas with the lowest mean scores were workforce development (score 1.3) and National Laboratory System with a score of 1.5 (Table 3). In the Respond category, the scores ranged from 1 to 2, with all the 14 indicators in the five technical areas having a score of 1.4 and below. The mean score was 1.2, and technical areas with the lowest mean score were preparedness, medical countermeasures and personnel deployment, and linking public health and security authorities with a score of 1 each (Table 4). In the other IHR-related hazards and point of entry category, all six indicators in three technical areas had a score of 1 each (Table 5).

**Table 2:** prevent category indicators score, 16-20 October 2017 the Republic of South Sudan

Technical areas	Indicators	Score
National legislation, policy and financing	P.1.1 legislation, laws, regulations, administrative requirements, policies or other government instruments in place are sufficient for implementation of IHR (2005)	Limited capacity-2
	P.1.2 the state can demonstrate that it has adjusted and aligned its domestic legislation, policies and administrative arrangements to enable compliance with IHR (2005)	No capacity-1
IHR coordination, communication and advocacy	P.2.1 a functional mechanism is established for the coordination and integration of relevant sectors in the implementation of IHR	Limited capacity-2
Antimicrobial resistance	P.3.1 antimicrobial resistance detection	No capacity-1
	P.3.2 surveillance of infections caused by antimicrobial-resistant pathogens	No capacity-1
	P.3.3 healthcare-associated infection (HCAI) prevention and control programs	No capacity-1
	P.3.4 antimicrobial stewardship activities	No capacity-1
Zoonotic diseases	P.4.1 surveillance systems in place for priority zoonotic diseases/pathogens	Developed capacity-3
	P.4.2 veterinary or animal health workforce	Limited capacity-2
	P.4.3 mechanisms for responding to infectious and potential zoonotic diseases are established and functional	No capacity-1
Food safety	P.5.1 the country has IBS or EBS and monitoring systems in place to monitor trends	Limited capacity-2
	and detect foodborne events (outbreak or contamination)	
Biosafety and biosecurity	P.6.1 whole-of-government bio-safety and bio-security system is in place for human, animal and agriculture facilities	No capacity-1
	P.6.2 biosafety and bio-security training and practices	No capacity-1
Immunization	P.7.1 vaccine coverage (measles) as part of a national program	No capacity-1
	P.7.2 national vaccine access and delivery	Developed capacity-3
Total score (N=15)		23
Mean score		23/15 (1.5)

**Note: 1(red)**=“attributes of a capacity do not exist or are not in place”; 2 (yellow)= “attributes of a capacity are in the development stage (some are achieved, and some are undergoing; however, the implementation has started)”; 3 (yellow) = “attributes of a capacity are in place; however, there is the issue of sustainability and measured by lack of inclusion in the operational plan in national health sector planning (NHSP) and/or secure funding”;4 (green) = “attributes are in place, sustainable for a few more years and can be measured by the inclusion of attributes or IHR (2005) core capacities in the national health sector plan-green”;5 (green) = “attributes are functional, sustainable and the country is supporting other countries in its implementation. This is the highest level of the achievement of implementation of IHR (2005) core capacities” (10)

**Table 3:** detect category indicators scores, 16-20 October 2021 the Republic of South Sudan

Technical Areas	Indicators	Scores
National laboratory system	D.1.1 laboratory testing for detection of priority diseases	2
	D.1.2 specimen referral and transport system	1
	D.1.3 effective modern point-of-care and laboratory-based diagnostics	2
	D.1.4 laboratory quality system	1
Real-time surveillance	D.2.1 indicator- and event-based surveillance systems	3
	D.2.2 interoperable, interconnected, electronic real-time reporting system	2
	D.2.3 integration and analysis of surveillance data	3
	D.2.4 syndromic surveillance systems	4
Reporting	D.3.1 system for efficient reporting to FAO, OIE and WHO	3
	D.3.2 reporting network and protocols in-country	2
Workforce development	D.4.1 human resources available to implement IHR core capacity requirements	1
	D.4.2 FETP or other applied epidemiology training program in place	1
	D.4.3 Workforce strategy	2
Total scores (N=13)		26
Mean score		26/13 (2)

**Table 4:** respond category indicators scores, South Sudan (October 16-20, 2017)

Technical areas	Indicators	scores
Preparedness	R.1.1 national multi-hazard public health emergency preparedness and response plan is developed and implemented	1
	R.1.2 priority public health risks and resources are mapped and utilized	1
Emergency response operations	R.2.1 capacity to activate emergency operations	1
	R.2.2 EOC operating procedures and plans	1
	R.2.3 emergency operations program	1
	R.2.4 case management procedures implemented for IHR relevant hazards.	2
Linking public health and security authorities	R.3.1 public health and security authorities (e.g. law enforcement, border control, customs) are linked during a suspect or confirmed biological event	1
Medical countermeasures and personnel deployment	R.4.1 system in place for sending and receiving medical countermeasures during a public health emergency	1
	R.4.2 system in place for sending and receiving health personnel during a public health emergency	1
Risk communication	R.5.1 risk communication systems (plans, mechanisms, etc.)	1
	R.5.2 internal and partner communication and coordination	2
	R.5.3 public communication	1
	R.5.4 communication engagement with affected communities	1
	R.5.5 dynamic listening and rumour management	2
Total score (N=14)		17
Mean score		17/14 (1.2)

**Table 5:** other international health regulation-related hazards and point of entry indicators score, South Sudan (October 16-20, 2017)

Technical areas	Indicators	Scores
Points of entry (PoE)	PoE.1 routine capacities established at points of entry	1
	PoE.2 effective public health response at points of entry	1
Chemical events (CE)	CE.1 mechanisms established and functioning for detecting and responding to chemical events or emergencies	1
	CE.2 enabling environment in place for the management of chemical events	1
Radiation emergencies (RE)	RE.1 mechanisms established and functioning for detecting and responding to radiological and nuclear emergencies	1
	RE.2 enabling environment in place for the management of radiation emergencies	1
Total score (N=6)		6
Mean score		6/6 (1)

## Discussion

The JEE of South Sudan, conducted in 2017 documented the country´s capacities to prevent, detect, and respond to public health threats per the IHR (2005) core capacity. The evaluators found the overall mean score of 48 indicators in 19 technical areas was 1.5 on a scale of 1-5. Out of 48 indicators, 30 (63%) showed the country has no capacity under the IHR (2005) requirement for health security. A total of 42 out of 48 indicators (88%) scored under the no capacity and limited capacity category. The outcome of JEE in South Sudan reaffirms the under-developed core capacities in all the 19 technical areas categorized in prevent, detect, respond, and IHR related hazards and points of entries. The low core capacities in place are similar to most African countries where JEE has been conducted [18]. A study conducted in 55 IHR states parties showed that 43 out of 48 indicators scored less than 4. Hence, countries in the WHO Regional Office for Africa (WHO-AFRO) performed poorly compared to countries in other regions [18,19]. In the ‘prevent’ category, the mean score was again 1.5 (no to limited capacity) 9 out of 15 (60%) indicators had a score of 1 (no capacity). The few areas with relatively well-developed capacities were mainly made by vertical programs, usually with external funding. For example, the vertical expanded program for immunization (EPI) national program is usually well resourced in human resources, cold chain, and training. The lowest score of no capacity was observed under antimicrobial resistance (AMR) and Biosafety/Biosecurity areas like most countries in the WHO-AFRO region due to lack of policy focusing in these areas [18,20]. At the same time, under the ‘Detect’ category, the score improved due to the investment in the Integrated disease surveillance and response (IDSR) system with accompanied reporting. The IDSR system has been robust enough to detect most outbreaks in South Sudan. The long years of constant investment in IDSR have paid the dividend [21,22]. Since the specific objectives of IDSR are to strengthen, coordinate, and streamline multiple disease surveillance activities to achieve an integrated, comprehensive public health surveillance system that serves all public health priorities at each level of the health system. This resulted in a strong IHR core capacities capacity in African countries that have implemented IDSR strategies over a long time [20].

In South Sudan, the health workforces have generally been low in numbers and skill mix. This is due to inadequate institution training, poor civil service remunerations, and high turnover. A similar finding was observed in the JEE; workforce development scored the lowest mean scores (i.e. 1.3). Countries across the WHO-AFRO region and worldwide faced a shortage of health workforce [19]. The health workforce is the foundation of the health system and essential to delivering quality health services, ameliorating population health, assuring universal health coverage (UHC), and attaining sustainable development goals (SDG). The 2013 world health assembly (WHA) and the ‘global strategy for human resource for health (HRH): workforce 2030’ acknowledge that health systems can perform well if they have sufficient, motivated, trained, responsive, competent and equitably distributed health workforce [23,24]. Countries in the WHO-AFRO region are expected to implement HRH strategy by 2030; however, implementation is lagging due to a lack of government commitment and health system investment [24]. As far as core capacities in the ‘respond’ category are concerned, they were all none or limited due to the underlying weak health system. This was manifested by weak coordination at the national level, limited community engagement due to a high level of illiteracy and meagre government resource investment into health which is less than 2% of gross domestic product (GDP) annually. In addition, the civil conflicts and the ongoing humanitarian situation added further stress to an already fragmented health system resulting in further decimation of the healthcare system [25]. Despite the challenges, the MOH, with support from partners, started constructing the public health emergency operation center (PHEOC). Once completed, it will drastically improve emergency response operations by providing strong coordination. Besides, during the JEE, review and completion of the national action plan for health Security (NAPHS) was underway. The NAPHS advocate for a multi-sectorial approach for better coordination of public health emergency preparedness and response at various levels.

For the category other IHR-related hazards and points of entry, the country had either no capacity or limited capacity as most other African countries [18]. Among the other IHR (2005) hazards, the country has a minimal ability to manage radiation and chemical events. As an oil-producing nation, South Sudan is at risk of chemical spills and thus, developing capacities in these technical areas is also critical [26]. The very low PoE score has far-reaching consequences of increased travel and trade between South Sudan and foreign countries. Because of the high volume of travelers, steps have been taken to strengthen ports health at Juba international airports and Nimule border crossing points. Our study shows that strong and participatory country self-assessment is critical to successfully implementing high-quality JEE and country ownership of its outcome. Furthermore, pilot site visits to selected national agencies were useful and substantially contributed to interpreting the objective and scoring of JEE technical areas. This facilitated collaboration between national officials and external experts [27]. Given the high burden of outbreaks and other public health emergencies, it is important the finding of JEE is used as it provides robust evidence to revise or develop the NAPHS [28]. The limitation of this study is that the scoring was done subjectively. At the same time, the participants from other non-health sectors did not entirely understand the scope of the questions. The knowledge of IHR (2005) was also limited among the participants, making it difficult for well-informed discussions.

## Conclusion

The policy implication of the findings of JEE is that the country must put in place plans and processes to progressively improve IHR core capacities in the context of health systems recovery. Our findings pointed to critical gaps in all the IHR (2005) core capacities and calls for urgent development and implementation of a NAPHS. Based on our findings, we propose the following recommendations. First, a mid-term review of the national IHR (2005) core capacities; proposed to assess ongoing efforts to fill the critical gaps identified during the study. Second, the country to finalize the NAPHS with a clear implementation framework. Besides, the country should take advantage of resources and partnerships available during acute emergencies such as the COVID-19 pandemic to improve some core capacities, particularly the NPHL and PHEOC.
